# Supplementary material for: Quantitative Evaluation of Stem-like Markers of Human Glioblastoma Using Single-Cell RNA Sequencing Datasets
Source: Cancers (Basel). 2023 Mar 2;15(5):1557. doi: 10.3390/cancers15051557 (PMC10001303; doi:10.3390/cancers15051557)
Supplement: Supplementary file 1 [file cancers-15-01557-s001.zip › cancers-2177473-supplementary/Supplementary Figures and Tables.pdf]

Figure S 1. Selection of markers based on the expression and the over-expression in stem-like subtype. The markers with top expressions are marked

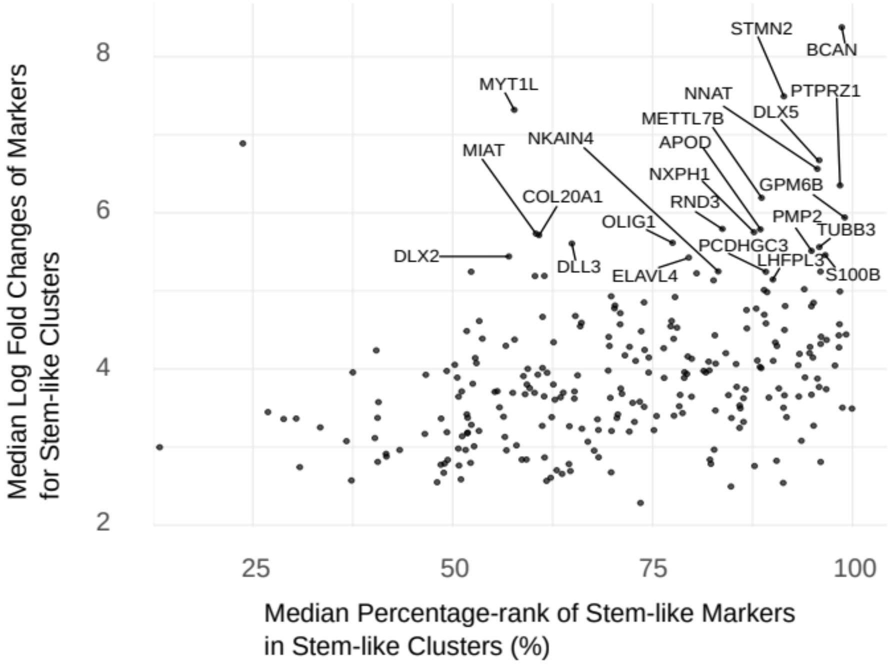

Figure S 2. Marker genes whose encoded proteins locate at membrane. Ploted with respect to frequency-significance selection

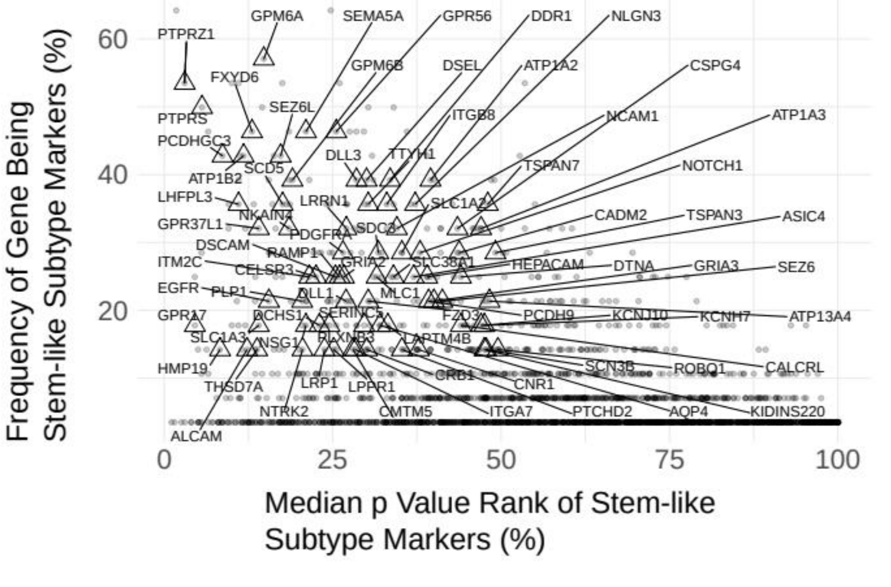

Table S 1. GBM samples and their corresponding names. The original names and the substituted names are listed in the same order

Study	Original names	Substituted names
Darmanis et al.	BT_S1, BT_S2, BT_S4, BT_S6	D1, D2, D3, D4
Patel et al.	MGH26, MGH28, MGH29, MGH30, MGH31	P1, P2, P3, P4, P5
Neftel et al.	BT749, BT771, BT786, BT830,	N1, N2, N3, N4
	BT920, BT1160, BT1187, MGH66,	N5, N6, N7, N8
	MGH85, MGH100, MGH101, MGH102,	N9, N10, N11, N12
	MGH104, MGH105, MGH106, MGH110,	N13, N14, N15, N16
	MGH113, MGH115, MGH121, MGH122,	N17, N18, N19, N20
	MGH124, MGH125, MGH128, MGH129,	N21, N22, N23, N24
	MGH136, MGH143, MGH151, MGH152,	N25, N26, N27, N28

Table S 2. GBM stem-like subtype - NPC1 cluster enrichment example (N20, cluster3)

GO:BP terms	term_id	adjusted_p_value
nervous system development	GO:0007399	3.65E-015
gliogenesis	GO:0042063	4.03E-11
glial cell differentiation	GO:0010001	5.89E-11
system development	GO:0048731	1.19E-10
central nervous system development	GO:0007417	5.73E-10
neurogenesis	GO:0022008	1.47E-09
regulation of nervous system development	GO:0051960	1.85E-09
anatomical structure development	GO:0048856	1.15E-08
multicellular organism development	GO:0007275	5.15E-08
cell adhesion	GO:0007155	5.36E-08

Table S 3. GBM stem-like subtype - NPC2 cluster enrichment example (N10, cluster 2)

GO:BP terms	term_id	adjusted_p_value
generation of neurons	GO:0048699	6.00E-17
neurogenesis	GO:0022008	7.85E-17
nervous system development	GO:0007399	2.34E-16
neuron differentiation	GO:0030182	4.08E-13
neuron projection development	GO:0031175	1.17E-10
neuron development	GO:0048666	2.97E-10
regulation of nervous system development	GO:0051960	3.90E-10
system development	GO:0048731	6.30E-10
cell development	GO:0048468	8.23E-10
regulation of neurogenesis	GO:0050767	2.48E-09

Table S 4. Overview of data analysis results, markers and their applications

Parameters	Corresponding figures	Recommended markers	Applications
1. universality/significance (high)	Figure 2b	BCAN, SOX4, GPM6A, PTPRZ1, SOX11, MAP2	Immunohistochemistry, in-vitro imaging, flow cytometry
1. universality/significance (high)<br>2. over-expression in GBM compared to normal cells (high)	Figure 2d	PTPRS, TUBB3, GPR56	In-vivo therapeutic targeting
1. universality/significance (high)<br>2. high expression level (high)	Figure 2b<br>Figure S1	BCAN, PTPRZ1, PMP2, GPM6B, TUBB3, S100B	In-vitro assays that require high expression level
1. universality/significance (high)<br>2. over-expression in GBM compared to normal cells (high)<br>3. high expression level (high)	Figure 2d<br>Figure 4	TUBB3	In-vivo targeting that requires a high expression level (such as in-vivo radionuclide-conjugated antibody targeting)
1. universality/significance (moderate)<br>2. over-expression in GBM compared to normal cells (moderate)<br>3. cell surface marker	Figure 2b<br>Figure 2d	PTPRS, ATP1A3, MAGED4, NNAT, ASIC4, ITGA7, GPR56, HMP19, LPPR1, MAGED4B, PTCHD2	In-vivo targeting that requires cell surface markers (such as targeted protein drugs)
1. universality/significance (high)<br>2. over-expression in GBM compared to normal cells (high)<br>3. high expression level (moderate)<br>4. cell surface marker	Figure 2b<br>Figure 2d<br>Figure 4	PTPRS, GPR56	In-vivo targeting that requires cell surface markers (such as targeted protein drugs)
